# Investigation of complications secondary to chest compressions before and after the 2010 cardiopulmonary resuscitation guideline changes by using multi-detector computed tomography: a retrospective study

**DOI:** 10.1186/s13049-017-0352-6

**Published:** 2017-01-26

**Authors:** Jin Ho Beom, Je Sung You, Min Joung Kim, Min Kyung Seung, Yoo Seok Park, Hyun Soo Chung, Sung Phil Chung, Incheol Park

**Affiliations:** 1 Department of Emergency Medicine, Yonsei University College of Medicine, 50 Yonsei-ro, Seodaemun-gu, 120-752 Seoul, Republic of Korea; 2 Department of Emergency Medicine, Hallym University Sacred Heart Hospital, Hallym University Medical Center, Gyeonggi-Do, Republic of Korea

**Keywords:** Cardiopulmonary resuscitation guideline, Chest compression, Complication, Multi-detector computed tomography

## Abstract

**Background:**

The purpose of this study was to identify the relationship between the deeper and faster chest compressions suggested by the 2010 cardiopulmonary resuscitation guidelines and complications arising from chest compressions, using multi-detector computed tomography.

**Methods:**

We performed a retrospective analysis of prospective registry data. This study was conducted with in- and out-of-hospital cardiac arrest patients who underwent successful resuscitation in the emergency departments of two academic tertiary care centres from October 2006 to September 2010 (pre-2010 group) and from October 2011 to September 2015 (post-2010 group). We examined chest injuries related to chest compressions, classified as follows: rib fracture, sternal fracture, and other uncommon complications.

**Results:**

We enrolled 185 patients in this study. The most frequent complication to occur in both groups was rib fracture: 27 (62.8%) and 112 (78.9%) patients in the pre-2010 and post-2010 groups, respectively (*p* = 0.03). However, we observed no statistical differences in sternum fracture, the second most common complication (*p* = 0.80). Retrosternal and mediastinal haematoma were not reported in the pre-2010 group but 13 patients (9.1%) in the post-2010 group were reported to have haematoma (*p* = 0.04). Nine serious, life-threatening complications occurred, all in the post-2010 group. Among the younger group (less than 65 years old), 8 (38.1%) patients in the pre-2010 group and 40 (64.5%) in the post-2010 group sustained rib fractures.

**Discussion:**

The deeper and faster chest compressions for enhancing ROSC are associated with increased occurrence of complications. Additional studies are needed to compensate for the limitations of our study design.

**Conclusions:**

This study found that the 2010 guidelines, recommending deeper and faster chest compressions, led to an increased proportion of rib fractures and retrosternal and mediastinal haematoma.

## Background

The cardiopulmonary resuscitation (CPR) guidelines issued in 2010 by the American Heart Association and the European Resuscitation Council recommend performing chest compressions at a speed of at least 100 compressions per minute and to a depth of at least 5 cm for adult patients with cardiac arrest [1, 2]. Compared with the 2005 guidelines, which recommended a rate of approximately 100 compressions per minute and a depth of approximately 1.5–2 inches (4–5 cm), the 2010 guidelines recommended increases in both parameters [3]. The faster and deeper chest compressions aim to improve the survival rate [4]. This emphasis on chest compressions is retained in the 2015 guidelines. The changes to the method of performing chest compressions have the potential to cause complications, [5] but there are few studies on this subject, and published study findings differ. Kralj et al. found that these changes had the biggest impact on increases in thoracic trauma frequency and the number of cases of thoracic trauma [6]. Hellevuo et al. reported that the deeper the chest compressions, the more complications occur [7]. On the other hand, Kashiwagi et al. reported that the incidence of complications did not increase significantly after introduction of the updated 2010 guidelines [8]. Most of the subjects in these studies were patients in whom a return of spontaneous circulation (ROSC) was not achieved, and a chest radiograph or autopsy result was used to diagnose complications [9]. A simple chest radiograph has limitations; it may miss subtle yet serious complications. Autopsy is a better tool, compared with chest radiography, for identifying a rib fracture; however, it has a lower sensitivity to identify partial rib fractures than does chest computed tomography [10–12]. Recently, studies using multi-detector computed tomography (MDCT) for detecting chest injuries secondary to CPR have been published. The usefulness of this technique has been demonstrated in several studies [13–15]. Thus, the purpose of this study was to identify the relationship between the deeper and faster chest compressions suggested by the 2010 cardiopulmonary resuscitation guidelines and complications arising from CPR, by using MDCT.

## Methods

### Study population and design

We performed a retrospective cohort study to identify whether deeper and faster chest compressions influence the rate of complications. This study was conducted with in- and out-of-hospital cardiac arrest patients who underwent successful CPR in the emergency departments of two academic tertiary care centres from October 2006 to September 2010 and from October 2011 to September 2015. The 1-year period from October 2010 to September 2011 was assumed a period of adjustment to the new 2010 version of the guideline; this period was thus excluded from the study period. Furthermore, we excluded patients aged less than 18 years, those who did not have chest MDCT within 48 h of ROSC, those who had a traumatic cause of cardiac arrest, and those transferred to the hospital after receiving CPR at another hospital. All patients in this study received CPR manually in- and out-of-hospital. In other words, no mechanical chest compression devices were used during the study period.

### Study protocol

The medical record of each patient included in the study was retrospectively analysed. We extracted data on age, sex, cause of arrest (cardiogenic/non-cardiogenic), witnessed or non-witnessed arrest, location where arrest occurred (in- or out-of-hospital), whether bystander CPR was provided, initial arrest rhythm, use or non-use of defibrillation and number of defibrillation attempts, number of adrenaline (epinephrine) doses given, and total CPR time. Chest MDCT was performed within 48 h after a successful CPR. All MDCT images were stored in the picture archiving and communication system (PACS, Centricity, GE Healthcare, Milwaukee, WI, USA). Complications were determined based on MDCT interpretation reports, performed and issued by each hospital’s board-certified radiologist.

We examined injuries sustained from performing CPR. Chest injuries related to chest compressions were classified as follows: rib fracture; sternal fracture; and other uncommon complications, such as lung contusion, lung haemorrhage, pneumothorax, haemothorax, retrosternal haematoma, and mediastinal haematoma.

Other extrathoracic complications secondary to chest compression were classified as follows: pneumoperitoneum, haemoperitoneum, scapula fractures, and vertebral fractures. In addition, rib fracture—the most common complication—was classified into four categories as follows: (1) Side of fractured ribs: one-side only, bilateral, or none; (2) Existence of multiple fractures; (3) Number of fractured ribs; and (4) Location of fractured ribs: anterior (from the parasternal line to the anterior axillary line), lateral (from the anterior axillary line to the posterior axillary line), or posterior (from the posterior axillary line to the paravertebral line) [16]. Sternal fractures were classified into those involving the proximal, middle, and distal sternum. The location of fractured ribs and the level of sternal fractures were assessed by one emergency physician using PACS. Of the injuries that occurred secondary to chest compressions, serious, life-threatening complications were defined as follows: haemoperitoneum or haemomediastinum from active bleeding, massive subcutaneous emphysema, pneumothorax with near total lung collapse, and pneumoperitoneum in conjunction with pneumothorax.

Subgroup analysis was conducted with variables identified by previous studies to influence the occurrence of CPR complications to determine whether there was a difference in complications before and after the guidelines changed. These variables were sex, location where the cardiac arrest occurred, and age [6–8]. The age variable was analysed dichotomised at 65 years of age [17]. Risk factors for increased complications of rib fracture and haematoma after changes to the guidelines were analysed by means of univariate and multivariable logistic regression.

### Statistical analysis

R version 3.2.1 (The R Foundation for Statistical Computing, Vienna, Austria) and SAS version 9.2 (SAS Inc., Cary, NC, USA) were used to perform the statistical analyses. Continuous variables were described as the mean ± standard deviation (SD), and categorical variables were described as frequencies (%). We used independent *t*-tests for comparison of continuous variables and Chi-square test or Fisher’s exact test for categorical variables, as appropriate. Variables with a *p* < 0.05 in the univariate analyses, and clinically significant variables (such as age and sex) proven in previous studies were selected as potential risk factors for CPR complications [6, 8, 18]. These variables were assessed using multiple logistic regression. Finally, the Mantel-Haenszel method was used to identify annual trend analysis of rib fracture after the guideline change. Statistical significance was defined as *p* < 0.05.

## Results

**Fig. 1:**
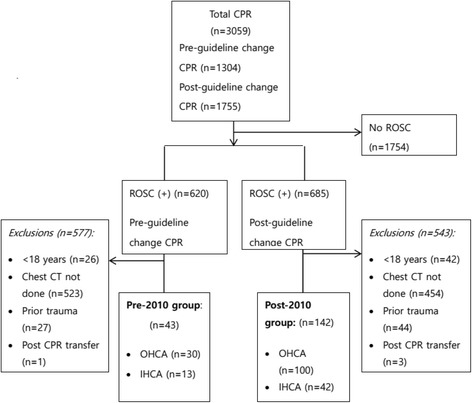
Flow diagram of patient eligibility. Pre-2010, pre-guideline change; Post-2010, post-guideline change; OHCA, out of hospital cardiac arrest; IHCA, in-hospital cardiac arrest; CPR, cardiopulmonary resuscitation; ROSC, return of spontaneous circulation; CT, computed tomography

During the study period, 3059 patients received CPR in the emergency departments of the two academic tertiary care centres. Of these, 1754 patients (57.3%) were excluded from the study because CPR was unsuccessful. Of the remaining 1305 (42.6%) patients, 620 (47.5%) and 685 (39.0%) achieved ROSC before and after the guideline was changed, respectively. Patients younger than 18 years, those who did not have chest MDCT within 48 h of ROSC, those who had a traumatic cause of cardiac arrest, and those who were transferred to the hospital after receiving CPR in another hospital, were excluded. Thus, 185 (6.0%) patients were enrolled for this study: 43 (30 out-of-hospital and 13 in-hospital) were included in the pre-2010 group and 142 patients (100 out-of-hospital and 42 in-hospital) were enrolled in the post-2010 group (Fig. 1, Table 1). The overall survival rates before and after the guideline changes were 1.9% (25/1304) and 3.6% (63/1755), respectively.

**Table 1:** Demographic characteristics and clinical findings

Variable	Pre-2010 (*n* = 43)	Post-2010 (*n* = 142)	*p*-value
Age (year)	63.2 ± 20.2	62.6 ± 17.2	0.86
Sex			0.11
Male	21 (48.8)	89 (62.7)	
Female	22 (51.2)	53 (37.3)	
Location of arrest			0.81
OHCA	27 (62.8)	92 (64.8)	
IHCA	16 (37.2)	50 (35.2)	
Bystander CPR	11 (25.6)	47 (33.1)	0.35
Initial rhythm			0.97
Shockable	4 (9.3)	13 (9.2)	
Non-shockable	39 (90.7)	129 (90.9)	
Number of defibrillation attempts	0.8 ± 2.6	0.7 ± 2.0	0.69
Number of doses of adrenaline given	2.7 ± 2.1	3.4 ± 2.6	0.11
CPR time (min)	20.8 ± 15.2	21.6 ± 15.6	0.77

Data are presented as mean ± standard deviation or n (%)

*Pre-2010* pre-guideline change, *Post-2010* post-guideline change, *OHCA* out of hospital cardiac arrest, *IHCA* in-hospital cardiac arrest, *CPR* cardiopulmonary resuscitation

The most frequent complication to occur in both groups was rib fracture: 27 (62.8%) and 112 (78.9%) patients in the pre-2010 and post-2010 group, respectively, sustained rib fractures (*p* = 0.03). The most second frequent complication was sternum fracture, which affected 13 (30.2%) patients in the pre-2010 group and 38 (26.8%) patients in the post-2010 group. However, this difference was not statistically significant (*p* = 0.80). Similarly, the groups did not differ significantly regarding the location of sternum fracture. Retrosternal and mediastinal haematomata were not reported in the pre-2010 group but 13 patients (9.1%) in the post-2010 group were reported to have haematomata (*p* = 0.04) (Table 2). Nine serious, life-threatening complications occurred, all in the post-2010 group. These complications included three cases of haemoperitoneum, two of which were related to active bleeding from the liver laceration and one of which was of unknown origin; haemomediastinum from active bleeding of the internal mammary artery (*n* = 1); massive subcutaneous emphysema (*n* = 2); tension pneumothorax (*n* = 1); and pneumothorax with extension to the pneumoperitoneum (*n* = 2), one of which was related to ascending colon perforation and one with an unknown origin, as shown in Table 2.

**Table 2:** Comparison of complications between before and after the change in the 2010 guideline

Variable	Pre-2010 (*n* = 43)	Post-2010 (*n* = 142)	*p*-value
Rib fracture	27 (62.8)	112 (78.9)	0.03
Multiple rib fracture	25 (58.1)	102 (71.8)	0.18
Number of rib fractures	4.2 ± 4.5	4.7 ± 3.8	0.47
Site of rib fracture			0.09
Unilateral	7 (16.3)	25 (17.6)	
Bilateral	20 (46.5)	87 (61.3)	
None	16 (37.2)	30 (21.1)	
Location of rib fracture			
Anterior	27 (62.8)	105 (73.9)	0.22
Lateral	17 (39.5)	73 (51.4)	0.23
Posterior	4 (9.3)	7 (4.9)	0.49
Sternum fracture	13 (30.2)	38 (26.8)	0.80
Location of sternum fracture			
Upper	5 (11.6)	6 (4.2)	0.15
Middle	6 (13.9)	26 (18.3)	0.67
Lower	6 (13.9)	20 (14.1)	>0.99
Lung contusion	7 (16.3)	35 (24.6)	0.35
Lung hemorrhage	4 (9.3)	16 (11.3)	0.93
Pneumothorax	4 (9.3)	25 (17.6)	0.28
Hematoma	0 (0)	13 (9.1)	0.04
Vertebral fracture	2 (4.6)	0 (0)	0.08
Scapular fracture	1 (2.3)	1 (0.7)	0.95
Serious complications	0 (0)	9 (6.3)	0.20
Massive subcutaneous emphysema	0 (0)	2 (1.4)	
Haemoperitoneum	0 (0)	3 (2.1)	
Haemomediastinum	0 (0)	1 (0.7)	
Tension pneumothorax	0 (0)	1 (0.7)	
Pneumoperitoneum	0 (0)	2 (1.4)	

Data are presented as mean ± standard deviation or n (%)

*Pre-2010* pre- guideline change, *Post-2010* post- guideline change

**Fig. 2:**
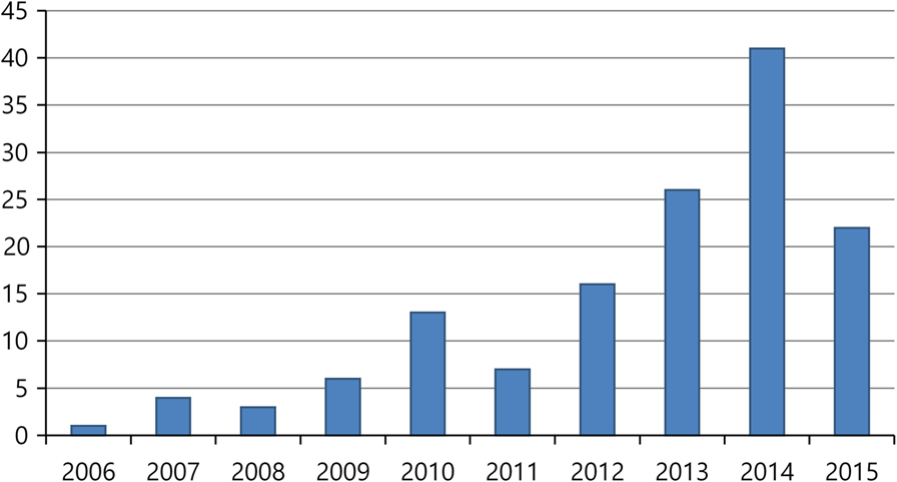
Annual trend analysis of rib fracture. X-axis: years. Y-axis: The number of patients who had rib fractures after CPR

The annual rate of rib fracture increased after the guidelines were changed. There was no significant difference in the annual rate of rib fracture occurrence from 2006 to 2009, but from 2011, a significant increase was observed (*p* = 0.02) (Fig. 2). In addition to the above analysis of complications, an analysis of the effect of the guideline changes on the occurrence of complications was conducted in specific groups. Results of these analyses are shown in Table 3. There was no significant difference in the occurrence of complications between the pre- and post-2010 groups according to sex, location of arrest, or age (when analysing the elderly group [greater than 65 years old]). However, among the younger group (less than 65 years old), 8 (38.1%) patients in the pre-2010 group and 40 (64.5%) in the post-2010 group sustained rib fractures (*p* = 0.03).

**Table 3:** Subgroup analysis of complications before and after the change in the 2010 guideline

	Pre-2010	Post-2010	*p*-value	Pre-2010	Post-2010	*p*-value
Variable	Male			Female		
Rib fracture	12 (57.1)	71 (79.8)	0.06	15 (68.2)	41 (77.4)	0.59
Multiple rib fracture	11 (52.4)	63 (70.8)	0.21	14 (63.6)	39 (73.6)	0.39
Sternum fracture	6 (28.6)	26 (29.2)	>0.99	7 (31.8)	12 (22.6)	0.59
Hematoma	0 (0)	5 (5.6)	0.60	0 (0)	8 (15.1)	0.13
Serious complication	0 (0)	4 (4.5)	>0.99	0 (0)	5 (9.4)	0.31
	OHCA			IHCA		
Rib fracture	19 (70.4)	78 (84.8)	0.16	8 (50.0)	34 (68.0)	0.32
Multiple rib fracture	18 (66.7)	70 (76.1)	0.48	7 (43.7)	32 (64.0)	0.15
Sternum fracture	11 (40.7)	28 (30.4)	0.44	2 (12.5)	10 (20.0)	0.76
Hematoma	0 (0)	8 (8.7)	0.20	0 (0)	5 (10.0)	0.33
Serious complication	0 (0)	7 (7.6)	0.35	0 (0)	2 (4.0)	>0.99
	Age < 65 y			Age ≥ 65 y		
Rib fracture	8 (38.1)	40 (64.5)	0.03	19 (86.4)	72 (90.0)	0.63
Multiple rib fracture	7 (33.3)	34 (54.8)	0.17	18 (81.8)	68 (85.0)	0.72
Sternum fracture	3 (14.3)	14 (22.6)	0.42	10 (45.4)	24 (30.0)	0.17
Hematoma	0 (0)	6 (9.7)	0.33	0 (0)	7 (8.7)	0.34
Serious complication	0 (0)	2 (3.2)	>0.99	0 (0)	7 (8.7)	0.34

Data are presented as n (%)

*OHCA* out-of-hospital cardiac arrest, *IHCA* in-hospital cardiac arrest

The univariate analysis examining risk factors for rib fracture and retrosternal or mediastinal haematoma, which increased in frequency after the 2010 guideline changes, identified older age, out-of-hospital CPR, and longer total CPR time to be associated with increased odds of rib fracture after the guideline was changed. These variables were included in the multiple logistic regression model. To exclude the correlation between the location of arrest variable and total CPR time variable, these two variables were analysed separately. Results from the analysis are described in Table 4. In terms of total CPR time, more rib fracture complications occurred after the guideline was changed (OR 3.31, 95% confidence interval [CI] 1.25–8.99, *p* = 0.02). Regarding the location of arrest, it was found that more rib fractures occurred when the patient was older (OR 1.08, 95% CI 1.05–1.11, *p* < 0.001), total CPR time was longer (OR 1.06, 95% CI 1.03–1.104, *p* = 0.001), and after the guideline changed (OR 3.02, 95% CI 1.19–7.85, *p* = 0.02).

**Table 4:** Multivariable logistic regression of associations with rib fracture

	Without CPR time		Without location	
Independent variables	OR (95% CI)	*p*-value	OR (95% CI)	*p*-value
Age	1.087 (1.06, 1.12)	0.06	1.079 (1.054, 1.109)	<0.001
Total CPR time			1.063 (1.028, 1.104)	0.001
Location of arrest	0.106 (0.032, 0.307)	0.77		
Before and after the change in the guideline	3.306 (1.252, 8.991)	0.02	3.024 (1.186, 7.847)	0.02

*OR* odds ratio, *CPR* cardiopulmonary resuscitation

## Discussion

This study found that the 2010 guidelines, recommending deeper and faster chest compressions, led to an increased proportion of rib fractures and retrosternal and mediastinal haematoma. The number of patients who had rib fractures tended to increase from 2006 to 2015. Excluding 2006 (when there was only 1 rib fracture case), before the guideline changes, the average rib fracture occurrence rate was 58%, rising to 79% from 2011 to 2015. It can be presumed that the gradual increase in the rib fracture rate from 2012 to 2014 may be correlated with user’s conformance to the updated 2010 guidelines. Our study attempted to raise confidence by excluding the 1-year period of adjustment directly after the change of guidelines. In contrast to rib fracture, we observed no statistical difference with regard to sternum fracture, the most second common complication in both groups, from before to after the guideline change.

The frequency of complications such as lung contusion, lung haemorrhage, and pneumothorax increased after the guideline changes, but these increases were not statistically significant. Furthermore, all of the nine cases of serious, life-threatening complications defined in this study (haemoperitoneum, haemomediastinum, massive subcutaneous emphysema, and tension pneumothorax) occurred only after the guidelines changed. The incidence of these serious complications was not very high. However, they might be life-threatening and might thwart resuscitation efforts [19, 20]. Hence, it is notable that these complications occurred only after the guideline was changed, suggesting that greater pressure was delivered to patients while performing chest compressions.

A subgroup analysis was performed to assess the impact of variables that previous studies had shown to influence CPR-related complications (sex, location of arrest, and age) on differences in the occurrence of complications pre- and post-2010 [6–8, 18]. The present study showed that there was no difference in the occurrence of complications before and after the guideline changes among elderly patients (>65 years old). However, there was an increasing proportion of rib fractures after the guidelines were changed among younger patients (<65 years old). This contrasts with findings of previous studies showing that, regardless of the changes to the guidelines, rib fractures after CPR occur more frequently among old patients than among young patients [6, 8, 21]. Regarding sex, it has been reported that women are more susceptible to CPR-related injuries compared with men [22–24].

However, in our study, there was no sex difference, even after institution of the 2010 guidelines. Likewise, no difference was found in the occurrence of complications after the changes to the guidelines between the in- and out-of-hospital groups. Similar to the findings of previous studies, the present study observed an increased proportion of rib fractures in patients who were older, had an out-of-hospital cardiac arrest, and for whom the total CPR time was longer [8, 11, 25, 26]. In addition, this study reported that the change of the guideline is a risk factor of rib fracture.

It was found that longer total CPR time is a risk factor for developing haematomata. However, since the haematoma variable had a frequency of zero before the change of the guideline, logistic regression methods could not be applied. Total CPR time was the only significant risk factor for haematoma, according to univariate analysis.

Previous studies found no major increase in the incidence of rib fracture, sternal fracture, and other serious complications before and after the changes to the 2010 CPR guidelines. However, most of these studies were conducted over a short period and were limited by the relatively small numbers of patients enrolled [8, 25]. A strength of the present study was the longer study period and greater number of patients enrolled.

However, the present study has a number of limitations. First, this study included only patients who achieved ROSC after CPR. Furthermore, only a subset of patients who achieved ROSC was included—those who underwent chest MDCT. During the study period, chest MDCT may have been performed according to the clinical requirement of the attending physician. However, it is difficult to determine the exact reason for performing chest MDCT because of the retrospective nature of the study. Hence, data from only 6.04% of all patients who received CPR were analysed. Second, it was a retrospective analysis conducted in only two medical institutions. Third, compared with the post-2010 group (*n* = 142), few patients who achieved ROSC in the pre-2010 group received chest MDCT (*n* = 43); hence, a relatively small number of patients was enrolled in the pre-2010 group. The performance of additional CT scans after guideline changes might be attributable to the recent trend in the medical field toward increased CT evaluation [14, 27]. However, the inter-group difference in the number of enrolled patients did not affect the outcome. Finally, autopsies are still considered the gold standard for investigating injuries associated with chest compressions. Several studies have demonstrated the usefulness of MDCT for detecting chest injuries secondary to CPR [13–15]. However, in a previous study, MDCT was reported to have lower sensitivity to detect complications related to CPR compared with autopsy [28]. Thus, the incidence of complications may have been underestimated in the present study.

## Conclusions

In this study, the overall survival rate was higher after the guideline change than before the guideline change, possibly as a result of the newly recommended faster and deeper chest compressions. However, the occurrence of complications such as rib fracture and retrosternal and mediastinal haematoma increased after the cardiopulmonary resuscitation guidelines were changed in 2010. A significant increase in the number of rib fractures was observed in patients younger than 65 years old. Serious, life-threatening complications occurred only after the guideline was changed. Therefore, the faster and deeper chest compressions for enhancing ROSC are associated with increased occurrence of complications. Additional studies are needed to compensate for the limitations of our study design, such as the very low number of included patients, and to determine how to balance the benefits and risks associated with the guideline changes.

## Abbreviations

CI: Confidence interval
CPR: Cardiopulmonary resuscitation
MDCT: Multi-detector computed tomography
OR: Odds ratio
PACS: Picture archiving and communication system
ROSC: Return of spontaneous circulation
SAS: Statistical analysis system
SD: Standard deviation
